# Genes and post-term birth: late for delivery

**DOI:** 10.1186/1756-0500-7-720

**Published:** 2014-10-14

**Authors:** William Schierding, Justin M O’Sullivan, José G B Derraik, Wayne S Cutfield

**Affiliations:** Liggins Institute, University of Auckland, Private Bag 92019, Auckland, New Zealand; Gravida: National Centre for Growth and Development, Auckland, New Zealand

**Keywords:** Developmental biology, Embryonic and fetal development, Epigenetic research, Genetic research, Parturition, Post-term birth

## Abstract

**Background:**

Recent evidence suggests that prolonged pregnancies beyond 42 weeks of gestation (post-term births) are associated with long-term adverse health outcomes in the offspring.

**Discussion:**

There is evidence that post-term birth has not only environmental causes, but also significant heritability, suggesting genetic and/or epigenetic influences interact with environmental cues to affect gestational length.

**Summary:**

As prolonged gestation is associated with adverse short- and long-term outcomes in the offspring, further research into the underlying genetic and epigenetic causes of post-term birth could be of importance for improving obstetric management.

## Discussion

Preterm (<37 weeks of gestation) and post-term (≥42 weeks of gestation) births can be described as the two ends of the gestational age continuum. However, there is a disparity in the amount of research allocated towards these ends of the gestation spectrum. There is considerable research on the short- and long-term effects of preterm birth, as well as into its potential underlying causes [1]. In contrast, although post-term birth is relatively common worldwide [2], it has attracted far less attention [3]. However, recent evidence suggests that prolonged pregnancies beyond 42 weeks are associated with long-term adverse health outcomes in the offspring [4, 5]. Importantly, there is evidence that while environmental stresses (*e.g.* malnutrition, insufficient myometrial contractility, intrauterine factors) play a large role in birth timing, over 25% of the variation in gestational age could be due to genetic effects [6]. More specifically, post-term birth has a large heritability, implicating genetics and/or epigenetics as contributing factors on gestational age [7–10].

### Post-term pregnancy: does an induced birth at 41 weeks count as a term pregnancy?

Post-term pregnancies are defined as those extending to or beyond 293 days (≥42 weeks) from the first day of the last menstrual cycle (assuming a 28-day cycle) [11]. However, classification of post-term birth can be affected by pregnancy complications and clinical uncertainty of menstrual dating [3]. Further, there are pregnancies that would become post-term without obstetric intervention, such as induction at 41 weeks of gestation. Prior to active management to prevent prolonged pregnancy, 19-20% of pregnancies were post-term when women were allowed to progress naturally to spontaneous labour [12]. ‘Best practice’ guidelines recommend induction of labour at 41 weeks [13–15], but clinical management varies [16] and approximately 2-5% of babies are born after 42 completed weeks of gestation [17]. Although induction reduces acute risks [13], it does not alter the underlying genetic influence on prolonged gestation and the associated risk profile. Therefore, we argue that the study of post-term birth should include ‘post-term potential’, i.e. those births that would have occurred post-term if left unmanaged. The implementation of modern induction practices has seen the post-term rate fall to under 5% of total births. Therefore, based on Pekkanen et al.’s data [12], as many as 15% of total term births could be classified as ‘post-term potential’. As such, 25% of routine inductions in low-risk pregnancies have prolonged pregnancy as the primary cause for induction [18, 19].

### What are the risks involved with post-term pregnancy?

Controversially, while epidemiological studies report a higher incidence of stillbirths and neonatal deaths in post-term pregnancies [3, 20], the only randomized controlled trial of intervention in prolonged pregnancy did not observe this difference [21]. Despite the controversy, post-term pregnancies are associated with many acute risks, including utero-placental insufficiency, fetal distress, non-progression, operative delivery (both operative vaginal and Caesarean), macrosomia, shoulder dystocia, low Apgar scores, and meconium aspiration [2, 22, 23]. Some studies have reported increased rates of post-partum haemorrhage following post-term delivery, especially in first pregnancies [24, 25], but others have not observed this association [26]. These acute risks lead to increased morbidity and mortality when compared to term infants. Perinatal morbidity and mortality reach a nadir in births at term (37–41 weeks), increasing as gestational age deviates further from term, for both preterm and post-term pregnancies [27].

Beyond the acute risks, there is limited but emerging evidence that post-term birth can have long-term adverse effects [4, 5, 28]. An adverse fetal environment, including fetal growth restriction (which can occur with post-term birth), has been associated with hypertension [29] and obesity in adulthood [30]. Recent small studies have found that post-term birth is associated with features of the metabolic syndrome in mid-childhood and obesity in mid-adolescence [4, 5]. In a cross-sectional study, Ayyavoo et al. found reduced insulin sensitivity, increased abdominal fat, impaired night-time blood pressure profile, and higher cholesterol levels in prepubertal children born post-term [4] with ultrasound confirmation of gestation. In a Swedish cohort followed longitudinally from birth to 16 years of age, post-term boys were found to be at higher risk of increased weight gain during childhood than term counterparts, leading to a greater incidence of obesity in adolescence [5]. As both of these are small studies, confirmation in larger cohorts is needed. Future research is necessary to better characterize the long-term health risks associated with post-term birth, informing and potentially influencing the obstetric management of prolonged pregnancies, particularly around the induction of labour.

### What epidemiological factors influence gestational age?

There are a number of maternal risk factors that have been found to be associated with an increased risk of post-term delivery. These include first-time birth, maternal age over 30 years, obesity, poorer education, lower socioeconomic status, ethnicity, and dietary factors (*e.g*. high omega-3 or docosahexaenoic acid intake during the last half of pregnancy) [10, 31–36]. The intake of common medicines can also affect gestational length, and low dose aspirin (a prostaglandin inhibitor) increases the likelihood of the pregnancy extending to post-term [37, 38].

### What is the evidence of a genetic effect on length of gestation?

Genetic factors explained 25-40% of the variation in birth weight, fetal growth rate, and gestational age among twins [6]. Chaudhari et al. discussed evidence of a genetic predisposition for preterm birth including recurrence of prematurity in mothers or across families, as well as an increased incidence in certain ethnicities [39]. Similarly, a history of post-term delivery in a previous pregnancy was found to be the most important risk factor for post-term births [40, 41], which was observed across a range of ethnicities [10]. Specifically, the risk of post-term delivery increases from 7.7%, to 19.9%, and 30% if the preceding birth was term, post-term, or extremely post-term (>44 weeks), respectively [42]. Moreover, sister pairs were more likely to experience post-term births than comparable brother pairs and brother-sister pairs, suggesting a possible transmission of genes down the mother-daughter line [7].

However, paternal genetics cannot be discounted, as the length of pregnancy differed by more than one week in families where there were different fathers between the index and subsequent birth [42]. This is further supported by the observation that if either the mother or father was born post-term, a child has an increased risk of also being post-term (49% and 23% greater, respectively), with further amplification of risk if both mother and father were born post-term [43]. When comparing maternal and fetal genetic influences on birth timing, fetal (26%) and maternal (21%) genetic factors explained almost half of the variation in gestational age, with environmental factors explaining the rest [7]. In twins, the heritability of post-term birth was estimated as 30%, with the association being greater for monozygotic as opposed to dizygotic twins, further implicating an underlying genetic (as opposed to environmental) influence [8].

To date there have been no studies examining the effect of any specific common gene variant on prolonged gestation. At the opposite end of the gestational age spectrum, the major genetic determinants of preterm birth (that could be used to diagnose or prevent prematurity) also have yet to be found [44], as a meta-analysis of the available studies has failed to support earlier associations of preterm birth with genetic loci [9, 45, 46]. These studies have included several single-nucleotide polymorphisms (SNPs) located within the follicle stimulating hormone receptor (FSHR) gene [47], a functional SNP in the promoter of the Serpin Peptidase Inhibitor, Clade H (Heat Shock Protein 47), Member 1 (SERPINH1) gene [48], and the type 1 insulin-like growth factor receptor (IGF1R) gene [49]. However, it has been shown that inclusion of very preterm infants (22–34 weeks) in the analysis by Lunde et al. resulted in a decrease in the estimated genetic effect on gestation, suggesting that the genetics of preterm, term, and post-term births may differ [9]. Therefore, while these studies indicate that the entire gestational age spectrum is influenced by genetics, the specific genes associated with preterm and post-term births could be entirely different.

### Beyond genetics: what are the effects of epigenetics on gestational age?

Transmissible phenotypes may not result solely from changes in the underlying DNA sequence. The epigenome can affect gene expression through DNA methylation, histone modification, chromatin folding, or small inhibitory RNA. Thus, alterations in DNA organization and packaging can enhance or suppress gene expression. For example, acetylation, phosphorylation, or methylation and ubiquitination of histone tails may alter the local chromatin structure, modifying the accessibility of the underlying DNA [50].

The epigenome is particularly susceptible to modification during gestation, neonatal development, puberty, and old age [51–54]. For instance, the relationship between epigenetics, aberrant nutrition, and lifetime metabolic dysfunction has been shown to have an effect in all of the above time periods, as well as having a trans-generational effect [55]. For post-term birth, the relevant time-period likely extends from conception until the end of gestation (i.e. the uterine environment), a period that is highly susceptible to epigenetic alterations [56]. For example, folate restriction in early gestation can cause global genome hypomethylation, restricting the ability of the fetus to impose cell-specific epigenetic modification [57].

Epigenetic changes in the fetus during pregnancy may affect length of gestation, with increased DNA methylation at three regions - near the nuclear factor I/X (CCAAT-binding transcription factor) (NFIX), Rap guanine nucleotide exchange factor (GEF) 2 (RAPGEF2), and methionine sulfoxide reductase B3 (MSRB3) genes - correlated with increased gestational age [58]. In addition, one study has found an association between LINE-1 DNA methylation (an inflammation marker) and preterm birth [59]. However, to date, no studies have examined potential epigenetic changes associated directly with post-term birth.

### Summary/future potential

Prolonged gestation is associated with adverse short- and long-term outcomes in the offspring. Evidence for the heritability of post-term birth suggests an important connection between birth timing and underlying genetic factors. This creates an important distinction where the genes involved in ‘post-term potential’ could be an underlying risk factor for later-in-life disease, despite the ‘best practice’ to induce at 41 weeks. This raises two possibilities: 1) the underlying genetic causes of prolonged gestation (post-term potential) are directly responsible for the adverse long-term health effects in post-term individuals; or 2) prolonged gestation itself leads to physiological alterations and associated epigenetic changes, which in turn lead to long-term adverse effects. Either way, a better understanding of the genetic and epigenetic factors leading to and/or resulting from post-term birth could explain the mechanism leading to long-term health risks. By understanding mechanisms underlying causation (as opposed to just clinical symptoms or outcomes), these risk prediction profiles could then form guidelines for alternative obstetric management (discussed for preterm birth in [60] and [61]). In the future, tests for genetic risk of post-term birth could inform obstetric treatment options earlier in pregnancy, possibly identifying those at risk for (or protected from) post-term birth. This could provide a basis for pharmacogenomic interventions that shorten gestation tailored to the individual’s genetic profile. Further research could also possibly lead to prolonged gestation being treated as a long-term effect, where both the acute and long-term consequences are taken into account and mitigated in a low-risk (to both baby and mother) and cost-effective manner.

## Abbreviations

SNP: Single-nucleotide polymorphism.
